# Association between CTSS gene polymorphism and the risk of acute atherosclerotic cerebral infarction in Chinese population: a case–control study

**DOI:** 10.1042/BSR20180586

**Published:** 2018-11-21

**Authors:** Lian Luo, Mingli Zhu, Jiajun Zhou

**Affiliations:** 1 Department of Neurology, Xixi Hospital of Hangzhou, Hengbu Street 2, Xihu District, Hangzhou, Zhejiang 310023, China; 2 Department of Clinical Laboratory, Xixi Hospital Of Hangzhou, Hengbu Street 2, Xihu District, Hangzhou, Zhejiang 310023, China; 3 Department of Neurology, Xixi Hospital of Hangzhou, Hengbu Street 2, Xihu District, Hangzhou, Zhejiang 310023, China

**Keywords:** Atherosclerosis, Cerebral infarction, Cathepsin S, Single nucleotide polymorphism

## Abstract

**Objective:** To investigate the association between the gene polymorphisms of rs774320676, rs768437857, rs928508030, and rs2275235 loci of Cathepsin S (CTSS) and risk of acute atherosclerotic cerebral infarction. **Methods:** A total of 315 patients with acute atherosclerotic cerebral infarction (study group) and 220 healthy subjects (control group) were enrolled in the present study. The genetic polymorphism of rs774320676, rs768437857, rs928508030, and rs2275235 loci of *CTSS* of subjects was analyzed by PCR-Sanger sequencing. **Results:** The proportion of carriers with mutant T allele at rs774320676 locus and mutant G allele at rs928508030 locus of *CTSS* in study group was significantly higher than the proportion in control group (*P*=0.000, adjusted odds ratio (OR) = 1.332, 95% confidence interval (CI) = 1.200–1.460; *P*<0.001, adjusted OR = 1.185, 95% CI = 1.055–1.314; *P*=0.002). The T allele at rs774320676 locus and the G allele at rs928508030 locus of *CTSS* were independent risk factors for acute atherosclerotic cerebral infarction (OR = 2.534, 95% CI = 1.020–4.652, *P*=0.006; OR = 2.016, 95% CI = 1.031–4.385, *P*=0.031). **Conclusion:** The single nucleotide polymorphisms (SNPs) of rs774320676 and rs928508030 of *CTSS* gene were related with risk for acute atherosclerotic cerebral infarction. The T allele at rs774320676 locus and G allele at rs928508030 locus of *CTSS* were genetic susceptibility genes of acute atherosclerotic cerebral infarction.

Cerebral infarction is a hypoxic and ischemic necrosis induced by insufficient blood supply of the local brain. It is caused by multiple factors, although atherosclerosis is the most common cause, and has a high morbidity rate and disability rate [1–3]. Atherosclerosis is the activation of the endothelium during the chronic inflammatory-fibrous proliferative response, which results in narrowing of blood vessels, insufficiency of cerebral blood supply, and secondary rupture of fibrous cap plaques, ultimately leading to cerebral infarction [4,5].

In recent years, the relation of genetic factors, especially gene polymorphism, and acute atherosclerotic cerebral infarction has become a hotspot in studies [6–8]. Cathepsin S (CTSS) is a cysteine protease of the papain superfamily, which plays an important role in extracellular matrix degradation and remodeling, antigen presentation, inflammation, immunity, and angiogenesis [9]. In recent years, studies have shown that CTSS plays an important role in the occurrence and development of atherosclerosis, plaque vulnerability, rupture, and the occurrence of clinical complications [10]. *CTSS* is located on chromosome 1, 1q21, and its protein is a cysteine protease of papain superfamily and plays an important role in the pathogenesis of chronic obstructive pulmonary disease (COPD) [11]. Some researchers found that CTSS gene polymorphisms are associated with obesity-related traits, and this association can be altered by dietary protein intake [12]. There is no relevant study on the impact of *CTSS* polymorphism on the morbidity risk of atherosclerotic cerebral infarction. In this study, single nucleotide polymorphisms (SNPs) at rs774320676, rs768437857, rs928508030, and rs2275235 loci of *CTSS* were selected for analysis. These mutations are either missense mutations or intronic mutations, and no reports have been reported yet.

## Materials and methods

### General information

Three hundred and fifteen patients with acute atherosclerotic cerebral infarction admitted to our hospital from August 2013 to December 2016 were selected as the study group in the present study, including 210 males and 105 females aged 52–73 years. All patients were diagnosed according to the diagnostic criteria of the International Classification of Diseases 10 (ICD-10) [13] and the classification standard of Trial of Org 10172 in Acute Stroke Treatment (TOAST) subtype [14]. Acute atherosclerotic cerebral infarction was diagnosed by Computed Tomography (CT) and MRI, and all patients are admitted to the hospital within 24 h of onset. Two hundred and twenty healthy people were enrolled as the control group, including 132 males and 88 females aged 54–72 years. Exclusion criteria: patients with malignancy or autoimmune disease; severe liver or kidney disease or blood disease; patients with heart failure, cardiomyopathy, atrial fibrillation, acute coronary syndrome, and other diseases; patients with thyroid disease; history of severe trauma or infectious disease; taking aspirin or clopidogrel 1 month prior to enrollment. The present study was approved by the medical ethics committee of our hospital, and all subjects signed the informed consent.

### Methods

#### Genome DNA extraction

Four millilitres of elbow vein blood of each fasting subject was extracted into an EDTA anticoagulant tube in the early morning. The blood genome DNA was extracted with QIAamp DNA Blood Mini Kit (QIAGEN, 51104, Germany) and was preserved at −80°C for further study.

#### Genotyping of SNPs

**Figure 1:**
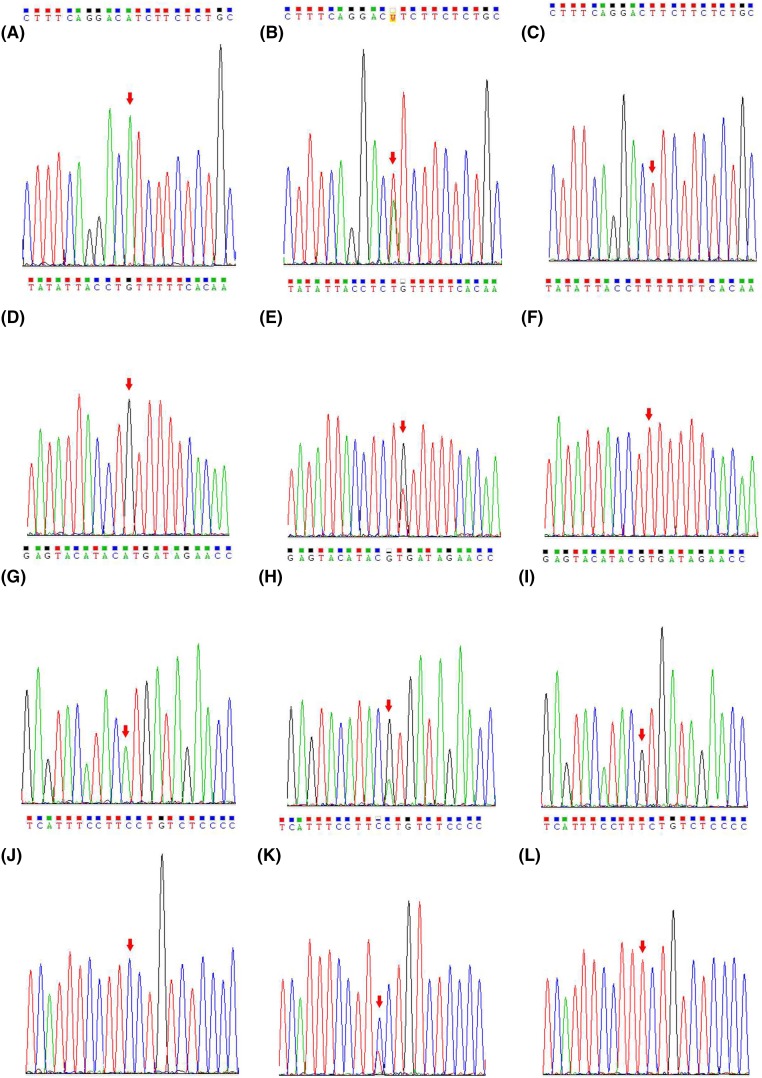
The results of Sanger sequencing of the CTSS SNPs loci (**A**) AA genotype at rs774320676 locus; (**B**) AT genotype at rs774320676 locus; (**C**) TT genotype at rs774320676 locus; (**D**) GG genotype at rs768437857 locus; (**E**) GT genotype at rs768437857 locus; (**F**) TT genotype at rs768437857 locus; (**G**) AA genotype at rs928508030 locus; (**H**) AG genotype at rs928508030 locus; (**I**) GG genotype at rs928508030 locus; (**J**) CC genotype at rs2275235 locus; (**K**) CT genotype at rs2275235 locus; (**L**) TT genotype at rs2275235 locus.

The PCR amplification primers were designed according to the sequence of rs774320676, rs768437857, rs928508030, and rs2275235 loci of the *CTSS* gene in the Ensembl database (Table 1). Preparation of PCR solution: 2.5 μl of 10× buffer, 1 μl of 25 mmol/l Mg^2+^, 0.5 μl of 10 mmol/l dNTP mix, 1 μl of each 10 μmol/l upstream and downstream primers, 0.25 μl of 5 U/μl Taq DNA polymerase, 1 μl of DNA template, and add water to make up to 25 μl. PCR conditions: 95°C predenaturation 4 min; 94°C denaturation 45 s, 56°C annealing 30 s; 72°C extension 30 s, and a total of 35 cycles with the last cycle at 72°C extension for 10 min. The PCR products were sequenced by Sanger sequencing (Figure 1).

**Table 1:** The PCR amplification primer information of *CTSS* gene SNPs loci

SNPs loci	Primer (5′→3′)	Annealing temperature
rs774320676	F: TACACCAACAGACACTGGGC;	58°C
	R: ATACTTCTTTTTGCAGGATCAGAAA	
rs768437857	F: ATTACCTGTTTTTCACAAGCCAGT;	59°C
	R: CATGGTGTACTTGTGGTTGGC	
rs928508030	F: AACCTAGCAGGCAGAACAAGT;	59°C
	R: AGGACTCTTACTGTGGGAGCA	
rs2275235	F: TTACCCAGACGTGAAAGTGGG;	59°C
	R: ATCTGGGCATGAACCACCTG	

### Statistical analysis

SPSS20.0 was used for statistical analysis. Measurement data were expressed as mean ± S.D. The difference between the two groups was compared by two groups of independent samples *t* test. The counting data were expressed as [*n*(%)]. Hardy–Weinberg equilibrium was calculated by χ^2^ goodness-of-fit test. The frequencies of genotypes and alleles were compared by the χ^2^ test, and the relative risk ratio was expressed as odds ratio (OR) and 95% confidence interval (CI). Multivariate logistic regression model was used to analyze the effect of genotypes and alleles of *CTSS* SNPS on the incidence of acute atherothrombotic cerebral infarction after adjustment for age, gender, alcohol consumption, smoking, Body mass index (BMI), and other factors. *P*<0.05 signified a statistically significant difference.

### Data availability statement

All data generated during and/or analyzed during the current study are available from the corresponding author on reasonable request.

## Results

### The comparison of general information

The general clinical information of the two groups is shown in Table 2. There was no significant difference in age, sex, BMI, drinking, triglyceride, total cholesterol, high-density lipoprotein, low-density lipoprotein, fibrin, and the number of patients with type 2 diabetes in the study group and the control group (*P*>0.05). The hypertension history, smoking ratio, systolic blood pressure, diastolic blood pressure, fasting blood glucose, and homocysteine of subjects in the study group were significantly higher compared with the control group (*P*<0.05).

**Table 2:** Comparison of general clinical data between the study group and the control group

Parameter	Study group (*n*=315)	Control group (*n*=220)	*t/χ^2^* value	*P*-value
Age (years, $\bar{\text{x}}\pm \text{s}$)	61.75±10.25	62.05±9.78	0.339	0.734
Gender [Male, *n*(%)]	218 (69.21%)	150 (68.18%)	0.063	0.801
BMI (kg/m^2^, $\bar{\text{x}}\pm \text{s}$)	23.35 ± 4.54	23.61 ± 4.19	0.662	0.508
Hypertension [*n* (%)]	226 (71.75%)	127 (57.73%)	11.3417.	0.001
Smoking [*n* (%)]	119 (37.78%)	40 (18.18%)	23.814	0.000
Drinking [*n* (%)]	75 (23.81%)	42 (19.09%)	1.688	0.194
Systolic pressure (mmHg)	156.54 ± 20.65	142.31 ± 19.87	7.965	<0.001
Diastolic pressure (mmHg)	88.54 ± 11.35	83.05 ± 12.04	5.369	<0.001
Triglyceride (mmol/l)	1.25 ± 1.22	1.21 ± 1.36	0.356	0.722
Fasting blood-glucose (mmol/l)	6.54 ± 2.21	5.74 ± 1.93	4.442	<0.001
High-density lipoprotein (mmol/l)	1.28 ± 0.41	1.31 ± 0.46	0.792	0.429
Low-density lipoprotein (mmol/L)	2.54 ± 0.81	2.49 ± 0.79	0.710	0.478
Fibrin (g/l)	3.49 ± 1.04	3.43 ± 1.54	0.503	0.615
Homocysteine (μmol/l)	14.76 ± 5.98	11.79 ± 5.42	5.872	<0.001
Type 2 diabetes [*n* (%)]	53 (16.83%)	44 (20.00%)	0.879	0.348

### Genotypes and allele frequencies of *CTSS* SNPs loci

The genotypes and allele frequencies of the *CTSS* SNPs loci in the study group and the control group are shown in Table 3. The genotype distribution of the SNPs loci of *CTSS* gene was in accordance with the Hardy–Weinberg balance (*P*>0.05) by the χ^2^ test. In the study group, the proportion of carriers with mutant T allele at rs774320676 locus and mutant G allele at rs928508030 locus was significantly higher than that in the control group (*P*=0.000, adjusted OR = 1.332, 95% CI = 1.200–1.460; *P*<0.001, adjusted OR = 1.185, 95% CI = 1.055–1.314; *P*=0.002). There was no significant difference in the genotypes and allele frequencies of rs768437857 and rs2275235 loci between the study group and the control group (*P*>0.05).

**Table 3:** Comparison of genotype and allele frequencies of the CTSS SNPs loci between the study group and the control group

SNPs	Study group (*n*=315)	Control group (*n*=220)	Crude OR (95% CI)	*P-*value	Adjusted OR (95% CI)	*P-*value
**rs774320676**						
Genotype						
AA	167	155	Ref			
AT	100	55	1.688 (1.116–2.555)	0.009	1.244 (1.049–1.453)	0.012
TT	48	10	4.455 (2.088–9.749)	<0.001	1.596 (1.315–1.802)	<0.001
Alleles						
A	434	365	Ref			
T	196	75	2.198 (1.610–3.002)	<0.001	1.332 (1.200–1.460)	<0.001
**rs768437857**						
Genotype						
GG	161	115	Ref			
GT	115	84	0.978 (0.664–1.439)	0.906	0.991 (0.740–1.162)	0.981
TT	39	21	1.327 (0.714–2.475)	0.340	1.114 (0.865–1.356)	0.419
Alleles						
G	437	314	Ref			
T	193	126	1.101 (0.835–1.451)	0.482	1.040 (0.927–1.157)	0.525
**rs928508030**						
Genotype						
AA	163	143	Ref			
AG	140	73	1.682 (1.154–2.455)	0.005	1.234 (1.061–1.25)	0.006
GG	9 (3.67%)	2 (1.37%)	2.764 (0.537–19.046)	0.184	1.408 (0.878–1.748)	0.149
Alleles						
A	466	359	Ref			
G	164	81	1.560 (1.144–2.129)	0.004	1.185 (1.055–1.314)	0.002
**rs2275235**						
Genotype						
CC	179	121	Ref			
CT	121	84	0.979 (0.671–1.429)	0.909	0.991 (0.847–1.153)	0.983
TT	15	15	0.60 (0.301–1.533)	0.312	0.840 (0.527–1.172)	0.414
Alleles						
C	479	326	Ref			
T	151	114	0.901 (0.674–1.206)	0.469	0.958 (0.842–1.078)	0.515

Factors such as age, sex, drinking, smoking, BMI, and other factors were adjusted by logistic regression in ‘ORa’.

### Multivariate logistic regression analysis of the risk factors associated with acute atherosclerotic cerebral infarction

Multivariate logistic regression analysis of the risk factors associated with acute atherosclerotic cerebral infarction is shown in Table 4. The data, such as the age, sex, BMI, hypertension history, smoking history, drinking history, blood pressure, diastolic blood pressure, fasting blood glucose, triglyceride, total cholesterol, high-density lipoprotein, low-density lipoprotein, fibrin, and homocysteine levels of subjects, the proportion of patients with type 2 diabetes, and frequencies of rs774320676 the T allele and rs928508030 G allele, were included in the regression equation. The backward elimination method was used for variable selection. Multivariate logistic regression analysis showed that systolic blood pressure, diastolic blood pressure, fasting blood glucose, homocysteine, diabetes, smoking history, and rs774320676 T allele and rs928508030 G allele of *CTSS* were independent risk factors of acute atherosclerotic cerebral infarction.

**Table 4:** Multivariate logistic regression analysis of risk factors associated with acute atherosclerotic cerebral infarction

Variables	Β value	Wals	*P*-value	*OR* (95%)
Systolic pressure	0.985	35.623	0.000	2.744 (1.581–4.593)
Diastolic pressure	0.572	14.256	0.034	1.812 (1.013–3.086)
Fasting blood glucose	0.962	31.653	0.001	2.612 (1.342–4.867)
Homocysteine	1.123	55.384	0.000	3.769 (1.982–4.325)
Diabetes	0.812	22.384	0.026	2.354 (1.024–4.751)
Smoking	0.891	26.157	0.001	2.561 (1.221–4.365)
rs774320676 T allele	0.815	23.413	0.006	2.534 (1.020–4.652)
rs928508030 G allele	0.754	19.875	0.031	2.016 (1.031–4.385)

Age (years): 0 = <55, 1 = 55–60, and 2 = >65; Gender: 0 = male, 1 = female; Hypertension: 0 = no, 1 = yes; Smoking: 0 = no, 1 = yes; Drinking: 0 = no, 1 = yes; Systolic pressure (mmHg): 0 = <140, 1 = ≥140; Diastolic pressure (mmHg): 0 = <90, 1 = ≥90; Triglyceride (mmol/l): 0 = <1.7, 1 = ≥1.7; Fasting blood-glucose (mmol/l): 0 = <7,1 = ≥7.0; Total cholesterol (mmol/l): 0 = <5.2, 1 = ≥5.2; High-density lipoprotein (mmol/l): 0 = <1.04, 1 = ≥1.04; Low-density lipoprotein (mmol/l): 0 = <2.58; 1 = ≥2.58; Fibrin (g/l): 0 = <4.0, 1 = ≥4.0; Homocysteine (μmol/l): 0 = <15, X = ≥15; Diabetes: 0 = no, 1 = yes; at rs774320676 locus: 0 = A allele, 1 = T allele; at rs928508030 locus: 0 = A allele, 1 = G allele.

## Discussion

Atherosclerotic cerebral infarction is a polygenic disease caused by the interaction of genetic and environmental factors. In the course of the disease, atherosclerosis can lead to stenosis and occlusion of the vascular cavity and thrombus formation, or a fall-off of unstable plaque that leads to cerebral infarction [15]. CTSS was only expressed in the fibrous cap and middle smooth muscle layer of atherosclerotic lesion, while it was not expressed in cultured vascular smooth muscle cells under normal conditions [16]. In a mouse model, researchers found that microvascular growth of *Ctss*^−/−^ mice was damaged after the loss of *CTSS*, indicating that CTSS plays an important role in extracellular matrix degradation and atherosclerotic plaque formation [17]. The expression of *CTSS* and *CTSK* (Cathepsin K) as well as their proteins in patients with atherosclerosis is significantly elevated, although the mechanism of CTSS in acute atherosclerotic cerebral infarction has not been studied yet. The present study investigated the role of CTSS in the development of atherosclerosis. Based on the role of CTSS in the development of atherosclerosis, we chose the *CTSS* rs774320676, rs768437857, rs928508030, and rs2275235 loci. At present, there are few studies on the relation between *CTSS* polymorphism and atherosclerosis. Therefore, we screened the Ensembl database for missense mutations or intron mutations.

From the results of the present study, it can be seen that the proportion of carriers with mutant T allele in rs774320676 locus and G allele in rs928508030 locus of *CTSS* in study group was significantly higher than in control group (*P*<0.05), indicating that the rs774320676 mutant T allele and the rs928508030 G allele of *CTSS* were risk factors for acute atherosclerotic cerebral infarction. These two allele carriers have a higher risk of acute atherosclerotic cerebral infarction than other gene carriers. However, there was no significant difference in genotype and allele frequency of rs768437857 locus and rs2275235 locus between the study group and the control group (*P*>0.05), indicating that the two SNP loci had no significant effect on the incidence of acute atherosclerotic cerebral infarction. Clinical studies showed that the level of CTSS is positively related with the degree of coronary atherosclerosis [18]. In addition, some animal studies have found that CTSS and its endogenous inhibitor CysC participate in the process of restenosis after balloon injury [19]. It can be seen that the relationship between rs774320676 and rs928508030 SNPs of *CTSS* gene and the acute atherosclerotic cerebral infarction is related with the CTSS protein. Whether this relation is reflected in the expression of *CTSS* gene or the function of CTSS protein still needs to be further studied. We believe that the *CTSS* rs774320676 mutation is a missense mutation, and amino acid sequence changes related to this mutation may affect the function of the expression. The rs928508030 mutation locus is located in the 5′ non-coding region and belongs to the regulatory region of protein expression. This mutation may affect the protein expression of *CTSS*, thus affecting the function of CTSS.

In addition, multivariate logistic regression analysis of risk factors related to acute atherosclerotic cerebral infarction showed that systolic blood pressure, diastolic blood pressure, fasting blood glucose, homocysteine, history of diabetes, smoking history, the T allele at rs774320676 site of *CTSS*, and the G allele at rs928508030 site of *CTSS* are independent risk factors of acute atherosclerotic cerebral infarction. The results of the present study can provide a theoretical basis for the prevention of acute atherosclerotic cerebral infarction in the clinical setting.

There are also some limitations in the results of the present study. First, there are few homozygous mutations in some SNPs, which affects the objectivity of the results and warrants the need for an increased sample collection. Second, acute atherosclerotic cerebral infarction is caused by multiple genes and factors, and so it is insufficient to only study several SNPs of *CTSS*. Thus, further studies on the synergistic effect of multiple genes and factors are needed.

## Conclusion

The rs774320676 and rs928508030 loci of *CTSS* gene are related to the risk of acute atherosclerotic cerebral infarction. T alleles at rs774320676 locus and G alleles at the rs928508030 locus are independent risk factors for acute atherosclerotic cerebral infarction.

## Abbreviations

CI: confidence interval
CTSS: Cathepsin S
OR: odds ratio
SNP: single nucleotide polymorphism
